# Differential Effects of Psychological Interventions in Online and Face-to-Face Settings on DSM-5 and ICD-11 Maladaptive Trait Domains: An Exploratory Pilot Study

**DOI:** 10.3389/fpsyt.2021.648367

**Published:** 2021-06-14

**Authors:** André Kerber, Carmen Schaeuffele, Tobias Krieger, Antoine Urech, Heleen Riper, Thomas Berger, Johanna Boettcher, Christine Knaevelsrud

**Affiliations:** ^1^Division of Clinical Psychological Intervention, Department of Education and Psychology, Freie Universität Berlin, Berlin, Germany; ^2^Department of Clinical Psychology and Psychotherapy, University of Bern, Bern, Switzerland; ^3^Department of Neurology, Inselspital Bern, Bern University Hospital, Bern, Switzerland; ^4^Department of Clinical, Neuro and Developmental Psychology, Vrije Universiteit, Amsterdam, Netherlands; ^5^Department of Research and Innovation, GGZ in Geest/Amsterdam University Medical Center, VU University Medical Center, Amsterdam, Netherlands; ^6^Psychologische Hochschule Berlin, Berlin, Germany

**Keywords:** PID5BF+, maladaptive traits, internet-based interventions for mental health, psychotherapy, DSM-5 AMPD, ICD-11 personality disorders

## Abstract

While mental health treatments have proven to be effective for a range of mental health problems, there is comparably little research on its effects on personality disorders or difficulty (PD). New dimensional conceptualizations of PD such as the ICD-11 PD model enable the cost- and time-effective dimensional assessment of severity and style of PD. Furthermore, they constitute a promising tool to investigate PD, not only as a treatment endpoint but also as a predictive or influencing factor for mental health treatments. In this study, we investigated the effects in two different mental health treatment settings [online (*N* = 38); face-to-face and blended [FTF/blended] (*N* = 35)] on the reduction of maladaptive personality traits as well as the interaction between maladaptive personality patterns and the response on primary endpoints (i.e., mental distress). Results indicate that both treatment settings have comparable within-group effects on the reduction of distress symptoms, while the treatment in the FTF/blended setting seems to have a stronger impact on the reduction of maladaptive traits. Further, reduction of maladaptive trait expressions was a reliable predictor of treatment response in the FTF/blended setting while explaining less variance in the online setting. Beyond the promising findings on the utility of maladaptive trait change as an outcome measure, we discuss possible applications as an information source for treatment decisions.

## Introduction

The ICD-11 includes a dimensional model for personality disorders (PD) comprising an assessment of severity of personality dysfunction and maladaptive personality traits or patterns (1). In the current and stable release, the ICD-11 defines five maladaptive personality trait domains: *Negative Affectivity* with core features such as emotional lability and poor emotion regulation, *Detachment* comprising limited emotional expression and intimacy avoidance, *Disinhibition*, e.g., impulsivity and irresponsibility, *Dissociality* centrally defined by self-centeredness and lack of empathy and *Anankastia* defined by perfectionism and rigidity. The ICD-11 PD model largely corresponds to the Alternative DSM-5 Model for Personality Disorders (DSM-5 AMPD) with respect to the 2-fold assessment of severity and style of PD, but somewhat differs in the definitions of maladaptive traits. While four of the five trait domains largely parallel (2), the DSM-5 model includes the domain of Psychoticism but lacks a separate trait domain for Anankastia. Recent studies therefore proposed measures assessing 6 maladaptive trait domains ensuring compatibility with both systems (3, 4). The DSM-5 AMPD has already accumulated a large body of research (5). This research not only endorses the reliability and utility of dimensional assessments of personality disorders but increasingly indicates that maladaptive personality patterns may constitute an important transdiagnostic factor for general psychopathology. Research showed moderate to strong associations of maladaptive personality patterns with a range of other mental disorders, e.g., anxiety and depression (6), internalizing and externalizing disorders (7), psychotic disorders (8), substance-related disorders (9), and posttraumatic stress disorder (10). They have also been shown to be related to transdiagnostic variables, such as interpersonal problems (11), childhood maltreatment (12), maladaptive schemas (13), pathological beliefs (14), emotion dysregulation (15) or attachment anxiety and avoidance (16).

These findings suggest that maladaptive personality patterns not only coincide with psychopathology but seem to play a central role as a perpetuating factor. This is in line with a longitudinal study that found a mutual reinforcement between neuroticism, negative life events, and (low) quality of life over the course of 16 years (17). Neuroticism is a personality trait highly correlated with ICD-11 Negative Affectivity and personality dysfunction [e.g., (18)]. Recent dimensional models of psychopathology go even further postulating that “the only systematic difference between symptoms and traits … is one of time frame” (19) with traits corresponding to more persistent features of psychopathology and symptoms being psychopathology features only manifest during specific time periods.

At the same time, personality traits seem to be amenable to change through clinical interventions. A recent meta-analysis investigated changes in personality traits across 207 clinical trials (20). The authors concluded that personality traits, most notably neuroticism and (low) extraversion, change through interventions with small to moderate effect sizes, and that these changes can already be achieved after 8 weeks of treatment. Due to considerable evidence that DSM-5 AMPD maladaptive traits can be conceived of as maladaptive variants of general personality traits (21), these may also be amenable through clinical intervention. Furthermore, the amount of change in maladaptive personality functioning seems to predict the long-term outcome of psychotherapy for depression (22) through a stronger resilience to negative life events. Recent theories of personality dysfunction therefore conceptualize PD as a “lack of resilience” (23). In line with the conceptualization of PD and personality problems as a lack of resilience is the high genetic, environmental and conceptual overlap between PD and insecure, disorganized or ambivalent attachment styles (24, 25). Both are centrally defined by enduring (dysfunctional) intra- and interpersonal regulation patterns and have a strong impact on general mental health (26, 27). Changes in these intra- and interpersonal regulation patterns through intervention may lead to better emotion regulation and disrupt the above described mutual reinforcement of psychopathology and negative life events.

Additionally, personality traits also seem to influence the course and outcome of mental health treatments, with more maladaptive expressions having a worse prognosis. In their review across 99 studies, Bucher et al. (28) found personality traits to be systematically related to psychotherapy outcomes with lower levels of neuroticism and higher levels of extraversion and agreeableness having the strongest impact, both on general improvement and the moderation of process variables such as working alliance. Similar findings were reported by Constantinou et al. (29), who found general and specific personality disorder factors to be differentially related to therapy course and outcome, with antisocial traits (low agreeableness) having the worst prognosis.

Taken together, maladaptive personality expressions seem to constitute a transdiagnostic and perpetuating factor for mental health issues, while at the same time being a predictive factor for treatment response and being amenable to change through clinical intervention.

This exploratory research study investigated the mutual interference of maladaptive personality patterns with two different settings of psychological treatments (online and face-to-face setting). We aim to explore the following questions: Are maladaptive personality patterns according to DSM-5 and ICD-11 amenable in the treatment settings investigated in this study? If so, how is this change associated with symptom response to psychological treatment? Are there differences regarding effects on maladaptive personality patterns between interventions in the online and face-to-face setting? Are certain maladaptive personality patterns predictive of treatment response in a given setting?

To these aims, we investigated indicators for maladaptive personality patterns according to DSM-5 and ICD-11 together with indicators for psychological distress at baseline and post-intervention measurement points in two different mental health settings.

## Materials and Methods

### Recruitment and Participants

#### Online Treatment Setting

For the internet-based treatment sample, data were collected within a randomized controlled trial on the efficacy of an internet-based version of the Unified Protocol (UP), a transdiagnostic intervention for internalizing disorders focused on emotion regulation processes, for patients with primary anxiety, depressive, or somatic symptom disorders (30). Participants received a 10-week therapist-guided internet-delivered intervention based on the UP, working self-paced through 10 modules with asynchronous (text-based) guidance once a week. This internet-based adapted version of the UP included all core interventions to target negative reactions toward emotions (understanding emotions, mindfulness, cognitive flexibility, countering emotional behaviors, and interoceptive, in sensu as well as *in vivo* exposures). The final sample consisted of 38 participants who completed treatment and had available data at baseline and post-treatment, i.e., 10 or 14 weeks post-randomization. For the subsequent analyses, we used the post-measurement in week 14, with missing data obtained by available data from week 10. Average age was 44.6 years (SD = 13.2, range = 23–66) with 24 (63%) participants being female.

#### Face-to-Face and Blended Treatment Setting

The second sample consisted of participants of the Swiss trial of the E-Compared study (31) comparing blended treatment, i.e., combining FTF sessions with an online tool, with treatment-as-usual (TAU). The TAU group received traditional FTF cognitive behavioral therapy [CBT; see (31)]. All participants were diagnosed by means of a MINI interview with a Major Depression Disorder (MDD). For the aims of the present study, participants from the two groups were combined since all participants were treated in outpatient departments of specialized mental health care settings. The final sample consisted of 35 participants with available data regarding measures under investigation at baseline and at 16 weeks post-randomization. Mean age of the sample was 40.1 years (*SD* = 15.5, range = 20–70), and 23 (65.7%) of the participants were female.

### Assessment Instruments

#### Maladaptive Trait Domains

##### Personality Inventory for DSM-5–Brief Form Plus

In the online setting, the PID5BF+ was assessed. The PID5BF+ is a 34-item self-report instrument based on the Personality Inventory for DSM-5 (PID-5), augmented with the ICD-11 personality trait domain Anankastia. The PID-5 is the official, 220-item self-report measure for the evaluation of maladaptive personality traits in five superordinate domains and 25 facets according to the DSM-5 AMPD (32, 33). The PID-5 has been extensively tested in clinical and non-clinical samples and has demonstrated adequate psychometric properties (5). DSM-5 AMPD maladaptive trait domains are compatible with 4 of the 5 maladaptive trait domains in the ICD-11 (34). The PID5BF+ assesses 17 maladaptive trait facets on a four-point Likert scale ranging from 0 = very false to 3 = very true that can be aggregated into 6 maladaptive trait domain scores—Negative Affectivity, Detachment, Antagonism, Disinhibition, Psychoticism (DSM-5 AMPD) and Anankastia (ICD-11 PD model). It is therefore compatible with both ICD-11 and DSM-5. Internal consistencies of PID5BF+ domain scale scores have been demonstrated to be adequate to high (4). For our subsequent analyses, we calculated the six maladaptive trait domain scores from the 17 averaged trait facet scores according to the scoring algorithm provided by Rek et al. (37). The average score of these six maladaptive trait domains can be used as an indicator for severity of personality dysfunction (subsequently PD severity) according to the DSM-5 section III and the ICD-11 PD model (35). The PID5BF+ was assessed in the online setting before and after the treatment.

##### Personality Inventory for DSM-5—Short Form

In the FTF/blended setting, the PID-5-SF was used. The PID-5-SF is an abbreviated 100-item version of the PID-5 with nearly identical psychometric properties (36). As above, for our subsequent analyses, we calculated the six maladaptive trait domain scores from 17 averaged trait facet scores according to the scoring algorithm provided by Rek et al. (37). The PID-5-SF was assessed in the FTF/blended treatment setting at baseline and at 16 weeks post-randomization. In a recent study, Rek et al. (37) found the PID5BF+ and PID-5-SF averaged domain scores to largely correspond to the same T-norms using multiple-group item response theory based on data of a nation-wide representative German population sample (*N* = 4,727).

#### Mental Distress

##### Brief Symptom Inventory

In the online setting, mental distress was assessed with the 18-item Brief Symptom Inventory [BSI-18; (38)], a valid and reliable self-report instrument of mental health symptom distress (39), rated on a 5-point-Likert scale assessing symptom burden ranging from “not at all” to “extremely.” The BSI-18 was assessed before and after the treatment.

##### Quick Inventory of Depressive Symptomatology

In the FTF/blended setting, mental distress was assessed with the Quick Inventory of Depressive Symptomatology [QIDS, (40)]. The QIDS is a reliable and valid 16-item self-report measure of the DSM-IV symptom criteria of MDD. Each item of the QIDS is scored from 0 to 3. The total score ranges from 0 to 27 because only the highest score is used from items that are components of a single DSM-IV criterion. The QIDS has demonstrated high levels of reliability and has been shown to be sensitive to change in several controlled studies. For the present study, we used the QIDS-scores assessed at 16 weeks post-randomization.

To assure comparability of the two different measures for mental distress, we calculated average instead of sum scores as both scales have a natural zero point and comparable descriptives in the two samples [BSI mean = 1.07 (range = 0–2.5) vs. QIDS mean = 1.28 (range = 0.11–2.67)].

Notably, average scores on baseline maladaptive traits and mental distress were not significantly different between participants with and without available post-treatment data in both samples except for Disinhibition in the online sample with non-completers having higher scores (*d* = 0.85 [CI = 0.31–1.38]).

### Statistical Analyses

#### Treatment Effects and Mean Differences of Maladaptive Trait Domains Between Treatment Conditions

To investigate whether maladaptive traits and mental distress changed over the course of treatment, we calculated Bonferroni-corrected paired *t*-tests. To assess the size of the difference effects, we calculated Cohen's *d* and 95%-confidence intervals of standardized mean differences using pooled standard deviations. To assess differences in maladaptive trait expressions and mental distress between the treatments at both measurement points, we calculated Bonferroni-corrected *t*-tests between trait domain and mental distress scores at the baseline and post measurement points.

#### Effects of Change in Maladaptive Trait Domain Expressions on Mental Distress

To investigate whether changes in maladaptive traits predicted post-treatment mental distress, we calculated regularized LASSO-regression (Least Absolute Shrinkage and Selection Operator) models with the baseline to post differences in 6 maladaptive traits plus PD severity and baseline mental distress score as independent variables and the post-assessment mental distress score as dependent variable using the *glmnet* R package (41). We calculated separate models for both settings to investigate whether the prediction of changes in maladaptive traits differed between both treatments.

Regularized or penalized regression models, such as LASSO-regression allow to account for high collinearity, i.e., non-independence of predictor variables. This seemed a suitable approach for 8 predictors of which seven are correlated maladaptive trait domains and one is the total average score of these maladaptive trait expressions. In their review including a comparison of methods to deal with collinearity, Dormann et al. (42) found LASSO regression to outperform other methods such as partial least squares regression in a dataset of 21 highly correlated predictor variables. Although regularized regression techniques such as LASSO existed already for decades in other disciplines, their utility for selecting predictor variables in the behavioral sciences gained visibility only recently (43).

In a second step, to ensure interpretability and comparability of the regression coefficients, we calculated a multiple regression model with post-treatment mental distress as the dependent variable including only predictors that yielded regularized regression coefficients differing from 0, i.e., that were > 0.001.

#### Maladaptive Trait Domains as Predictors of Treatment Response

To evaluate whether maladaptive trait expression at baseline may be a useful information source for decisions concerning the selection of treatment modalities, we calculated multivariate LASSO-regression models with the reduction of mental distress in either of the two treatments, i.e., two standardized pre-to-post difference scores, one per treatment, as dependent variables. As above, in a second step, we calculated a multiple regression model only including predictors that survived the LASSO regression procedure.

## Results

### Treatment Effects and Mean Differences of Maladaptive Trait Domains Between Treatment Conditions

**Figure 1:**
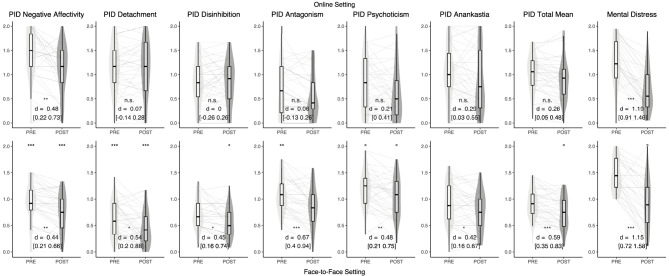
Combined violin- and boxplots depicting distribution, median and standard deviations of maladaptive personality traits and mental distress. Gray lines are individual data. The upper row depicts data from the online mental health treatment condition, the lower row depicts data from the face-to-face/blended. Signs between the upper and lower row indicate significance of between-treatment differences, signs between boxplots represent within-treatment pre-post differences. °uncorrected *p* < 0.05, *Bonferroni corrected *p* < 0.05, **Bonferroni corrected *p* < 0.01, ***Bonferroni corrected *p* < 0.001.

Figure 1 depicts mean differences of maladaptive traits and mental distress at baseline and post-treatment measurement-points for both settings.

Mental distress levels were comparable both at the beginning and the end of the treatments but with differences in the assessment measures impeding the interpretation of significance tests. Both treatments had comparably strong pre-to-post effects on psychological symptomatology.

In the online setting, a pre-to-post difference in maladaptive trait domain expressions was only found for Negative Affectivity. Conversely, all 6 maladaptive trait domains as well as the total average score showed significant change in the FTF/blended setting.

Maladaptive trait domain expressions at the beginning of the treatments were found to be significantly higher for Negative Affectivity, *d* = 1.18 [1.05–2.43] and Detachment, *d* = 1.22 [0.99–2.25] in the online setting, while Antagonism, *d* = 0.82 [0.47–1.75], was significantly higher in the FTF/blended setting. At post-treatment, Negative Affectivity, *d* = 0.99 [0.74–2.07], Detachment, *d* = 1.51 [1.40–2.68] and Disinhibition, *d* = 0.68 [0.24–1.30] were still higher in the online setting. Psychoticism was moderately higher in the FTF/blended setting at baseline (*d* = 0.64 [0.17–1.11]) with a smaller difference at post-treatment (*d* = 0.49 [0.01–0.96]) but in both cases with significance not surviving Bonferroni correction.

At the beginning of treatment, the personality dysfunction severity was comparable in both settings. In the online setting, the mean of 1.03 corresponds to a T-Score of 58.2 (35), in the FTF/blended setting, the mean of 0.92 corresponds to a *T*-Score of 55.6. After treatment, there were moderate but not statistically significant differences in total average maladaptive trait scores between the treatment conditions with 0.92 [*T* = 55.6] in the online setting and 0.73 [*T* = 53.5] in the FTF/blended setting, corresponding to *d* = 0.48 [0.01–0.95].

### Effects of Change of Maladaptive Trait Domain Expressions on Mental Distress

For both settings, the results of the regularized LASSO-regression revealed only the pre-to-post difference in PD severity and the baseline mental distress score to account for a significant proportion of variance (see Table 1). Accordingly, we omitted the remaining difference scores in trait domains from the subsequent multiple linear regression model.

**Table 1:** Results of LASSO regularization and multiple regression models.

		**Post treatment mental distress score**							**Treatment response** **(Δ** **mental distress baseline to post treatment)**					
		**Internet-based setting**			**FTF/blended setting**				**Internet-based setting**			**FTF/blended setting**		
		**LASSO regression**	**Multiple regression**		**LASSO regression**	**Multiple regression**			**Mutivariate LASSO regression**	**Multiple regression**		**Multivariate LASSO regression**	**Multiple regression**	
**Predictor**	**Score**	**Regularized coefficient**	**β**	**ΔR^**2**^**	**Regularized coefficient**	**β**	**ΔR^**2**^**	**Score**	**Regularized coefficient**	**β**	**ΔR^**2**^**	**Regularized coefficient**	**β**	**ΔR^**2**^**
Negative affectivity	Δ	–	–		–	–		PRE	0.117	0.25^**^	0.08	−0.078	−0.29^**^	0.10
Detachment	Δ	–	–		–	–		PRE	0.032	0.20^*^	0.05	−0.018	−0.10	0.01
Disinhibition	Δ	–	–		–	–		PRE	–	–		–	–	
Antagonism	Δ	–	–		–	–		PRE	−0.005	−0.20^*^	0.06	0.008	0.16	0.03
Psychoticism	Δ	–	–		–	–		PRE	–	–		–	–	
Anankastia	Δ	–	–		–	–		PRE	–	–		–	–	
PID total mean	Δ	−0.012	−0.41^**^	0.10	−0.534	−1.29^***^	0.49	PRE	–	–		–	–	
Mental distress	PRE	0.284	0.54^***^	0.42	–	0.52	0.16	PRE	0.013	0.11	0.02	0.032	0.31^**^	0.11
R^2^		–	53.0%		–	55.3%			–	33.0%			28.3%	

* *p < 0.05*,

** *p < 0.01*,

*** *p < 0.001, two-tailed*.

* Standardized regression coefficient (β). Change in R^2^ if predictor is removed from the regression (ΔR^2^). Change between baseline and post-treatment assessments (Δ). Baseline assessment (PRE). Least Absolute Shrinkage and Selection Operator (LASSO). Regularized LASSO-Regression coefficients were calculated using cross-validation to find optimal λ value for the most regularized model such that error is within one standard error of the minimum. Treatment response was calculated for each treatment separately*.

The multiple linear regression model predicting post-treatment mental distress in the online setting by the pre-to-post change in PD severity and the baseline mental distress score revealed both indicators to have a significant impact on the post-treatment mental distress level [*F*_(2, 35)_ = 19.8, *p* < 0.001, *R*^2^ = 0.53], with the baseline mental distress score explaining a larger proportion of the post mental distress variance (*R*^2^ = 0.42) than the PD severity difference (*R*^2^ = 0.10).

Predicting the post-treatment mental distress score in the FTF/blended setting by the pre-to-post difference in PD severity and the baseline mental distress score, revealed both indicators having a strong impact on post-treatment mental distress [F_(2, 32)_ = 19.8, *p* < 0.001, *R*^2^ = 0.55]. Here, the pre-to-post difference in personality dysfunction explained the larger proportion of post-treatment mental distress reduction (*R*^2^ = 0.49) in comparison to the baseline mental distress score (*R*^2^ = 0.16).

### Maladaptive Trait Domains as Predictors of Treatment Response

The LASSO-regularized multivariate regression model predicting treatment response to one of the two treatments based on baseline maladaptive trait expressions yielded Negative Affectivity, Detachment, Antagonism and baseline mental distress levels to be differentially predictive of treatment response in the two treatment settings (see Table 1). Multiple linear regression predicting post-treatment mental distress in the online setting by baseline scores on these predictors revealed higher Negative Affectivity and Detachment levels and lower Antagonism scores to be predictive for a larger reduction of mental distress in the online setting [F_(4, 68)_ = 8.4, *p* < 0.001, *R*^2^ = 0.33]. In the FTF/blended setting, multiple linear regression predicted a stronger reduction of post-treatment mental distress for lower Negative Affectivity and higher mental distress at baseline [F_(4, 68)_ = 6.7, *p* < 0.001, R_2_ = 0.28].

## Discussion

The exploratory results of the present study showed that both treatments had comparably large within-group effects on the reduction of mental distress while differing in their effects on maladaptive personality traits. More precisely, change in maladaptive trait domains through mental health treatment seems to be possible in both settings, at least with respect to the self-reported maladaptive traits scores in the two study samples. In the online setting, only Negative Affectivity showed a significant change. This is in line with a central postulation of the used treatment rationale of the Unified Protocol which was explicitly developed to target neuroticism and has been shown to elicit changes on neuroticism (44). Furthermore, the result is comparable to Johansson et al. (45) also investigating personality trait change in an internet-based intervention, who found significant effects only for neuroticism. In the FTF/blended setting, despite the depression-focused and CBT-based treatment rationale, all six maladaptive trait domains as well as PD severity, showed significant change. While the severity of personality dysfunction was comparable in the two settings at baseline, the between-treatment difference was larger when comparing the post-treatment measurements. While changes on all trait dimensions are in line with previous research on personality trait change through clinical interventions, in contrast to Roberts et al. (20), effect sizes in the FTF/blended setting were comparable for all trait domains in the present study.

Secondly, most predictive for treatment response was not the change in single trait domains such as Negative Affectivity but the reduction of total PD severity. This finding is in line with previous research findings that severity of personality dysfunction seems to predict future comorbidity better than specific PD styles, i.e., maladaptive traits (46). Furthermore, the reduction of the total personality dysfunction score was a more central predictor of post-treatment mental distress reduction in the FTF/blended (49% ΔR^2^) than in the online (10% ΔR^2^) treatment setting. Change in maladaptive trait domains seems therefore to be differentially intertwined with reduction in mental distress depending on the treatment setting. Although the results of this exploratory study must be interpreted with caution, an explanation for these treatment-bound differing impact factors and the stronger reduction of maladaptive personality patterns in the FTF/blended setting could be the inherently interpersonal nature of both traditional FTF psychotherapy and maladaptive personality patterns. Maladaptive personality traits are consistently associated with generalized interpersonal dysfunction (11) and etiologically closely related to attachment experiences (25). Traditional FTF psychotherapy seems to have a strong impact on interpersonal problems (47), independent of the theoretical orientation, with the therapist's interpersonal skills being a reliable predictor for treatment outcome (48). Following these previous findings, it seems more likely that maladaptive (interpersonal) personality patterns, e.g., devaluating others to regulate self-worth, restricting affect communication and intimacy to avoid rejection, or being extremely clingy to avoid separation, often leading to recurring adverse life events, social reinforcement loss and distress, need process-based interactional experiences to change. Another important issue for further research may therefore be to find ways to quantify the centrality of interpersonal dysfunction in individual psychopathologies, as only individuals with interpersonal dysfunction centrally perpetuating their psychopathology may need those direct-interactional-focused treatments. Besides interactional treatment components, targeting “functional mechanisms through which an individual's personality confers risk for psychopathology” with specific CBT techniques and interventions seems to be a promising and effective way for future (internet-based) treatment conceptualizations (49).

Thirdly, while baseline scores of Negative Affectivity were positively associated with the reduction of mental distress in the online setting, this association was negative for mental distress reduction in the FTF/blended setting. This finding may partly be explained by the specific neuroticism-addressing emotion-regulation components of the internet-based UP treatment making it more suitable for individuals with high negative affectivity. On the other hand, baseline Antagonism scores were negatively associated with mental distress reduction in the online setting and higher baseline mental distress levels were only positively associated with mental distress reduction in the FTF/blended setting. An explanation for this finding may be that personality factors associated with worse outcome such as disagreeableness (28) can be addressed more specifically in FTF settings, as more individualized treatment settings seem to lead to better treatment outcomes, especially for individuals with more symptom severity (50). Another likely explanation may be a selection bias in the two samples due to the lack of randomization. This latter explanation is also in line with the findings concerning Detachment. While higher Detachment scores were positively associated with response in the online treatment condition, individuals seeking online treatment had on average significant higher scores in Detachment compared to individuals in the FTF/blended treatment condition. Though it is likely that interventions in a pure online setting may be more attractive for people avoiding intimacy and/or social contacts, the question if there is a self-selection bias of individuals with higher Detachment in online interventions needs to be answered in future research.

The present study has several limitations. First, our results and conclusions should be interpreted with caution due to small sample sizes. Sample sizes in both settings, as well as the combined sample, are too small for reliable inferences considering the mostly moderate effect sizes. Additionally, allocation to treatment settings was not randomized implying selection-bias concerning baseline scores. Second, both mental distress and maladaptive traits were assessed differently in the two samples leading to systematically differing variances impeding between-treatment comparisons. Variables were assessed in different ways in the two samples because they originate in larger previous studies that were planned and implemented independently. Third, we only analyzed the completer sample, implying a further selection effect, though baseline-differences between completers and non-completers were negligible. Fourth, we had no follow-up data to investigate if reduction in maladaptive personality patterns leads to a better prognosis for long-term remission. Fifth, in the FTF/blended setting, average treatment duration was 16 weeks compared to 10 weeks in the online setting. Consequently, the difference in personality pattern changes may also only be due to a longer treatment.

Despite these limitations, our results preliminarily substantiate previous research identifying maladaptive personality patterns to play an important role, both as transdiagnostic factors as well as for the prognosis and indication of mental health treatments. Assessing the severity and style of personality dysfunction according to ICD-11 may therefore not only be a valid instrument for the diagnosis of PD but also an indicator of the degree and duration of process-based interactional experiences that an individual patient may need to profit in the long-term of a treatment. However, the question whether individuals with certain maladaptive traits such as Antagonism benefit more from treatments in the FTF setting than in an online setting is a question for further research. Future research also needs to address whether change in personality functioning leads to higher resilience and a reduced risk for relapse in the long-term.

## Data Availability Statement

The data analyzed in this study is subject to the following licenses/restrictions: Data can be obtained upon reasonable request from the corresponding author. Requests to access these datasets should be directed to andre.kerber@fu-berlin.de.

## Ethics Statement

The studies involving human participants were reviewed and approved by Ethikkommission des Fachbereiches Erziehungswissenschaft und Psychologie, Freie Universität Berlin and Kantonale Ethikkommission Bern. The patients/participants provided their written informed consent to participate in this study.

## Author Contributions

AK analyzed the data and wrote the initial draft, which was then edited and reviewed by CS, TK, AU, HR, TB, JB, and CK. TK, TB, AU, and HR were responsible for the data acquisition in Switzerland. CS, JB, CK, and AK were responsible for the data acquisition in Germany. All authors contributed to the article and approved the submitted version.

## Conflict of Interest

The authors declare that the research was conducted in the absence of any commercial or financial relationships that could be construed as a potential conflict of interest.
